# Supplementary material for: The Association of IFNL4 Gene Polymorphisms with Hepatitis B Virus (HBV) Infection in the Northern Region of Pará, Brazil
Source: Int J Mol Sci. 2024 Oct 9;25(19):10836. doi: 10.3390/ijms251910836 (PMC11476639; doi:10.3390/ijms251910836)
Supplement: Supplementary file 1 [file ijms-25-10836-s001.zip › ijms-3099946-supplementary.pdf]

SUPPLEMENTARY MATERIAL

Table S1. Sociodemographic characterization of people with HBV.

IFNL4	**rs12979860**
	**n=69 (%)**
**Biological sex**	
Female	27 (39%)
Male	42 (61%)
**Self-declared ethnicity**	
White	3 (4.3%)
Black	8 (11.6%)
Brown	21 (30.5%)
Uninformed	37 (53.6%)
**Age (years)**	
24 - 29	4 (6%)
30 - 39	15 (22%)
40 - 49	21 (30%)
50 - 59	11 (16%)
≤ 60	18 (26%)
**Residence**	
Metropolitan Region of Belém, Pará	49 (71%)
Pará's countryside	18 (26%)
Another Brazilian state	1 (1.5%)
Uninformed	1 (1.5%)

n = Number of total people considered.

Table S2. Paired comparison (Fisher's exact test) of different genotypes and clinical manifestations.

IFNL4	**Allele Genotype**	**Symptomatic, n=11 (%)**	**Asymptomatic, n=35 (%)**	**Symptomatic vs. Asymptomatic**
**rs12979860**	TT + TC	9 (82%)	29 (83%)	p=0.9873
	CC	2 (18%)	6 (17%)	
	T	45%	53%	
	C*	55%	47%	

* Minor allele frequency (MAF).

Table S3. Paired comparison between chronic HBV vs. control groups (Chi-squared test).

IFNL4	**Allele Genotype**	**Chronic HBV, n=43 (%)**	**Control, n=85 (%)**	**Chronic HBV vs. Control**
**rs12979860**	TT + TC	35 (81%)	53 (52%)	p=0.1592
	CC	8 (19%)	32 (38%)	
	T	51%	38%	
	C*	49%	62%	

* Minor allele frequency (MAF).

Table S4. Paired comparison of the different *IFNL4* genotypes and the levels of HBV DNA, AST/TGO, ALT/TGP, and GGT (Mann–Whitney).

	IFNL4			
	**rs12979860**			
	**TT**	**TC**	**CC**	**TT+TC vs. CC**
**HBV DNA (UI/mL)**	174*	987.5*	427*	p=0.7646
**AST/TGO (U/L)**	30*	30.35*	26.5*	p=0.3271
**ALT/TGP (U/L)**	29*	32*	29.5*	p=0.2922
**GGT (U/L)**	35*	31.5*	25*	p=0.1781

*Median value.
